# High prevalence of malaria in a non-endemic setting: comparison of diagnostic tools and patient outcome during a four-year survey (2013–2017)

**DOI:** 10.1186/s12936-018-2218-4

**Published:** 2018-02-05

**Authors:** Adriana Calderaro, Giovanna Piccolo, Sara Montecchini, Mirko Buttrini, Sabina Rossi, Maria Loretana Dell’Anna, Valeria De Remigis, Maria Cristina Arcangeletti, Carlo Chezzi, Flora De Conto

**Affiliations:** Department of Medicine and Surgery, University of Parma, Viale A. Gramsci, 14, 43126 Parma, Italy

**Keywords:** Malaria, Diagnosis, Tools, Therapy, Outcome, Epidemiology, Immigrants, Italy

## Abstract

**Background:**

Malaria is no longer endemic in Italy since 1970 when the World Health Organization declared Italy malaria-free, but it is now the most commonly imported disease. The aim of the study was to analyse the trend of imported malaria cases in Parma, Italy, during January 2013–June 2017, reporting also the treatment and the outcome of cases, exploring the comparison of the three diagnostic tests used for malaria diagnosis: microscopy, immunochromatographic assay (ICT) (BinaxNOW^®^) and Real-time PCR assays detecting *Plasmodium falciparum, Plasmodium vivax, Plasmodium malariae*, *Plasmodium ovale curtisi, Plasmodium ovale wallikeri*, and *Plasmodium knowlesi*.

**Results:**

Of the 288 patients with suspected malaria, 87 were positive by microscopy: 73 *P. falciparum*, 2 *P. vivax*, 8 *P. ovale*, 1 *P. vivax*/*P. ovale*, 1 *P. malariae* and 2 *Plasmodium* sp. All samples were positive by ICT except 6. Plasmodial DNA was revealed in the 87 cases and in 2 additional cases showing *P. falciparum*-specific bands by ICT, as follows: 75 *P. falciparum*, 2 *P. vivax*, 6 *P. ovale curtisi*, 3 *P. ovale wallikeri*, 1 *P. malariae*, and 2 mixed infections. 72 patients were foreigners and 17 Italians travelling for tourism or business. The majority of these patients presented with fever at blood collection and did not have chemoprophylaxis. No fatal cases were observed and the drug mostly used was quinine observing a negative blood smear or a parasitaemia < 0.001% after 48–72 h’ therapy.

**Conclusions:**

The study shows an update and a thorough analysis of imported malaria cases in the area of Parma during 4.5 years from the point of view of the total case management, clinical and diagnostic. The prevalence of malaria in such area in the considered period was especially due to immigrants mostly from Africa. Molecular methods were more sensitive and specific than microscopy and ICT, both detecting additional cases of *P. falciparum* malaria missed by microscopy and correctly identifying the Plasmodium species of medical interest. The data reported in this study may stimulate the clinicians in non-endemic areas to suspect malaria also in cases, where the most typical symptoms are absent, and the parasitologists to confirm the results of microscopy, remaining the reference method, with molecular methods to avoid misdiagnosis.

## Background

Imported malaria is described as “an infection acquired in an endemic area, but diagnosed in a non-endemic country after development of the clinical disease” [1]. The increasing number of international travels and the significant influx of immigrants from malaria-endemic countries had a significant impact on malaria cases in developed countries. Most of imported malaria cases are subjects travelling to visit friends and relatives in their countries of origin followed by visitors and new entrants from abroad, as well as travellers for holiday or business [2]. The confirmed malaria case rate in Europe in 2014 was 1.24 cases per 100,000 population, the highest in 2010–2014 [3]. Nearly all of the reported malaria cases were imported, 5 were locally acquired: 2 in France and 3 in Spain (1 congenital *Plasmodium falciparum* malaria, 1 induced *Plasmodium malariae* infection in a patient with a kidney transplant and 1 introduced *Plasmodium vivax* infection). Malaria is a mandatory notifiable disease in Italy; according to the data of the Ministry of Health [4], during 2011–2015, 3633 cases of malaria (89% with a confirmed diagnosis) were notified, most of which imported and 7 indigenous (2 induced, 3 cryptic, 1 suspected ‘baggage’ case and 1 suspected introduced case); *P. falciparum* was the mainly detected species and the deaths were 4.

Early and accurate diagnosis and prompt treatment of clinical malaria are essential for reducing malaria morbidity and mortality, drug resistance and malaria control. The primary objective of the treatment is to ensure the rapid and complete elimination of the *Plasmodium* parasite from the patient’s blood in order to prevent progression of uncomplicated malaria to severe disease or death, and to prevent chronic infection that leads to malaria-related anaemia [2]. In cases of misdiagnosis or failure in the detection of *Plasmodium* sp. by microscopy, the treatment could be delayed or inappropriate [5].

Three common methods are used to diagnose malaria: microscopic examination of Giemsa-stained thick and thin blood smears, rapid diagnostic tests (RDTs), and Polymerase Chain Reaction (PCR) or other nucleic-acid based assays [6, 7].

As reported by the World Health Organization (WHO) [2], microscopy remains the mainstay of parasite-based diagnosis in most large health clinics and hospitals, the quality of microscopy-based diagnosis is frequently inadequate for ensuring good sensitivity and specificity of malaria diagnosis. It is well-known that microscopy is an imperfect gold standard [8], because its accuracy is related to innate ability, training, experience, motivation, and laboratory resource. Thus, results are operator-dependent and in countries with imported malaria, maintaining the expertise of all microscopists seems to be the main problem. Persons suspected of having malaria, but whose blood films do not indicate the presence of parasites, should have blood films repeated every 12–24 h for a total of 3 sets. If films remain negative, then diagnosis of malaria is essentially ruled out [9] and anti-malarial therapy should be avoided [2]. The other methods for malaria diagnosis, RDTs and PCRs, are not yet validated in the clinical trial setting [2].

RDTs have been developed to detect antigens derived from malaria parasites; such tests often use a dipstick or cassette format, and provide results in 2–15 min offering a useful alternative to microscopy in situations where reliable microscopic diagnosis is not immediately available [10]. These assays identify parasite antigens such as HRP2, pLDH and pAldolase [2, 11] and are routinely implemented due to their simplicity and rapid turnaround time, enabling them to work as a point-of-care diagnostic. Although there are many types of RDTs, with varying degrees of reliability and accuracy [12], there is currently only one, BinaxNOW^®^, the only United States Food and Drug Administration-approved RDT for malaria, that qualitatively detects both the histidine-rich protein 2 (HRP-2), specific to *P. falciparum*, and aldolase, a pan-malarial antigen found in all *Plasmodium* species [2, 13]. However, malaria RDTs from different manufacturers can present wide variations, especially in terms of performance characteristics, and can be affected by sub-optimal transport or storage conditions [14]. Currently available devices have also some limitations: batch-to-batch quality variation, species and density determination, persistent positivity, accuracy, and costs [12]; moreover, false negative results in the presence of high parasitaemia are reported [12]. The specificity of RDTs can be low, particularly in the setting of persistent antigenaemia post-treatment of *P. falciparum* infection, as well as non-*falciparum* species [10], although newer generation RDTs perform well in diagnosing *P. vivax* infections [15]. For all these reasons RDTs may be used in addition to, but not as a replacement for microscopy [15].

The molecular detection of malaria parasites is highly accurate and sensitive, and remains the best method for detecting multiple species and sub clinical infections [6, 16]. The barriers to adoption of PCR include its high cost and the amount of infrastructure required in terms of equipment and a sophisticated laboratory setup with stable power and refrigerators for reagent storage [17]. Thus, its availability is limited to well-resourced reference facilities. PCR malaria detection has been based on several different target genes [18] but the majority of molecular diagnostic tools still rely on 18S rRNA gene because it is specific, conserved in all *Plasmodium* species, and has about 5 to 7 copies per *Plasmodium* genome, improving detection sensitivity [6, 19, 20]. Real-Time PCR assays have high sensitivity and specificity, and they are used to confirm ambiguous microscopic results and to identify mixed infections [6, 20].

The aim of this study is to analyse the trend in malaria cases at the University Hospital of Parma, Italy, a non-endemic area, during January 2013-June 2017 reporting also the treatment practices and the outcome of malaria cases, exploring the performances of the three diagnostic tests routinely used in our laboratory to diagnose malaria: microscopy, immunochromatographic assay (BinaxNOW^®^ Malaria) and Real-time PCR assays able to detect *P. falciparum*, *P. vivax*, *P. malariae*, *Plasmodium ovale curtisi*, *P. ovale wallikeri* and also the species *Plasmodium knowlesi* in subjects native or coming from Southeast Asia, endemic area for this species [2, 6].

## Methods

### Study area and population

The study was performed at the University Hospital of Parma, a 1137-bed tertiary care centre with more than 47,656 admissions registered in the year 2016, while access to Emergency Unit amounts to 112,933 [21] and a surrounding area of about 448,000 inhabitants at January 2017 [22]. The population attending this hospital was estimated in 207,594 inhabitants, about 10% of whom were immigrants, mainly from developing countries [23] including malaria endemic areas.

### Patients

This study was conducted from January 2013 to June 2017 and enrolled 288 patients coming from endemic areas for malaria and presenting on admission at the University Hospital of Parma with signs and symptoms consistent with malaria, mostly chills, fever and sweating, headache, anaemia, splenomegaly. Clinicians were contacted to know some information about patients, using an appropriate questionnaire entitled “Malaria Urgency” prepared in the laboratory following the instructions of the Ministry of Health and duly completed at the time of blood sample arrival in the laboratory for each patient with suspected malaria: date of birth, nationality, recent travels through endemic areas for malaria, intake of anti-malarial chemoprophylaxis, clinical signs and symptoms, presence of fever at the time of blood sampling.

### Laboratory diagnosis

Each blood sample, containing at least 5 ml of blood drawn into sterile tubes with EDTA, arrived in the laboratory within 1 hour from the collection and immediately used to prepare thin blood films for microscopic observation; the blood smears were stained with acridine orange and with 1% Giemsa in phosphate-buffered saline (pH 7.0), according to standard procedures [6, 20, 24], and examined for the research of *Plasmodium* sp. by fluorescent microscope (400× and 1000× magnification) and by light microscope (1000× magnification), respectively. Smears were examined and were defined as negative if no parasites were observed in the whole film. Identification and count of *Plasmodium* sp. was done on Giemsa-stained smears: parasites concentration (parasitaemia) was calculated by counting the number of parasitized red blood cells seen in 10,000 erythrocytes on a thin-film and expressing the number as a percentage, as described for non-endemic areas [11, 20, 24].

Blood samples were also submitted to the immunochromatographic assay (ICT) (BinaxNOW^®^Malaria) for the qualitative detection of the Histidine-Rich Protein II (HRPII) antigen specific to *P. falciparum* and a pan-malarial antigen common to *P. vivax/P. ovale/P. malariae*, following manufacturers’ instructions.

An aliquot of each blood sample was also submitted to real-time PCR assays to confirm the microscopic diagnosis. Genomic DNA was purified from each blood samples as previously described [6, 20, 24]; according to the results of microscopic examination (negative or positive), purified DNAs were subjected to a genus- or to species-specific Real-time PCR assays able to detect *P. falciparum/P. vivax/P. ovale curtisi/P. ovale wallikeri/P. malariae*/*P. knowlesi*, respectively, all targeting the 18S ribosomal RNA gene of the genus *Plasmodium* [6, 20, 24]. No samples were examined for the presence of *P. knowlesi* since no patients were natives or came from Southeast Asia.

An aliquot of DNA of each sample was stored at − 20 °C for further examination. Each PCR run included a negative control (a reaction mixture without DNA), and 6 positive controls consisting of plasmids containing DNA oligonucleotides with the target sequence of *Plasmodium* sp., *P. falciparum*, *P. malariae*, *P. vivax*, *P. ovale curtisi*, *P. ovale wallikeri* ssrRNA gene (synthesized by TIB Molbiol S.r.l., Genova, Italy), respectively. A *P. falciparum* positive blood sample confirmed by sequencing [6] was alternatively used as positive control before the plasmid control purchase. Each blood sample negative for *Plasmodium* sp. was submitted to a Taq-Man based Real-Time PCR assay specific for the human β-actin gene as previously described [6], in order to assess both the success in DNA extraction and the absence of inhibitors of the DNA polymerase.

## Results

For 87 (30.2%) of the 288 subjects with suspected malaria included in the study, *Plasmodium* sp. were detected by microscopy: 73 *P. falciparum*, 2 *P. vivax*, 8 *P. ovale*, 1 *P. vivax*/*P. ovale*, 1 *P. malariae* and 2 *Plasmodium* sp. (Table 1). The parasitaemias ranged from < 0.001% to 15.7% (Table 1). The remaining 201 subjects were negative for the presence of malaria parasites in the blood by microscopy.

**Table 1:** Microscopy, ICT and Real-time PCR results of samples from patients with suspected malaria

Microscopy		Parasitaemia		ICT		Real-time PCR assays	
No.	Species	No.	Range (%)	No.	Result	No.	Species/result
73	*Pf*	72	<0.001**–**15.7	72	T1 alone or T1 + T2	72	*Pf*
		1	0.65	1	T1 + T2	1	mixed *Pf* + *Po*
2	*Pv*	2	0.17**–**0.21	2	T2	2	*Pv*
8	*Po*	1	0.62	1	T2	1	*Poc*
		3	0.12**–**0.21	3	Negative	3	*Poc*
		1	0.40	1	T2 (low intensity)	1	*Pow*
		1	0.20	1	T2	1	*Pow*
		1	0.17	1	T2	1	*Pm*
		1	0.09	1	Negative	1	*Pow*
1	*Pv*/*Po*	1	0.25	1	T2 (low intensity)	1	*Poc*
1	*Pm*	1	< 0.001	1	Negative	1	mixed *Pf* + *Pm*
2	*P.* sp.	1	< 0.001	1	T1	1	*Pf*
		1	0.26	1	Negative	1	*Poc*
2	NA	**–**		2	T1	2	*Pf*
199	NA	**–**		199	Negative	199	Negative

NA, not applicable because the result was negative; *Pf, P. falciparum; Pv, P. vivax*; *Poc, P. ovale curtisi*; *Pow*, *P. ovale wallikeri*; *Pm*, *P. malariae*; *P*.sp., *Plasmodium* species

ICT, Immunochromatographic assay (BinaxNOW^®^ Malaria): the appearance of T1 band alone or T1 + T2 band indicates a *Pf* only or a mixed infection of *Pf* with another species; the appearance of T2 band only indicates a *Pv/Po/Pm* infection

The 73 *P. falciparum* positive samples by microscopy showed malaria specific antigens by ICT (presence of T1 alone or both T1 and T2 bands) (Table 1); 2 *P. vivax* positive samples showed the presence of only T2 band, 4 *P. ovale* positive samples showed the presence of only T2 band (in one of these cases with low intensity), 1 *P. vivax*/*P. ovale* showed a T2 low intensity band, 1 *Plasmodium* sp. positive sample showed the presence of *P. falciparum* specific band (T1). The other 5 positive samples (4 *P. ovale*, 1 *P. malariae* and 1 *Plasmodium* sp.) by microscopy showed no bands by ICT. Two out of the 201 negative samples by microscopy showed *P. falciparum* specific bands and the remaining 199 negative blood samples showed no plasmodial specific bands (Table 1).

For 89 (30.9%) of the 288 subjects with suspected malaria, the presence of plasmodial DNA was revealed by species-specific Real-time PCR (75 *P. falciparum*, 2 *P. vivax*, 6 *P. ovale curtisi*, 3 *P. ovale wallikeri*, 1 *P. malariae*, 1 mixed infection *P. falciparum* + *P. ovale* and 1 *P. falciparum* + *P. malariae*) (Table 1); the remaining 199 samples were negatives.

The prevalence of imported malaria in our area in the considered period was 30.9% and the prevalences per year were as follows: 29.1% in 2013, 31.5% in 2014, 37.5% in 2015, 32.8% in 2016, and 33.3% from January to June 2017.

Among the overall 89 patients with imported malaria, 64 (71.9%) were foreigners, including 1 Romanian and 1 Greek visiting Ivory Coast and Sudan, respectively, 17 (19.1%) were Italians travelling for tourism or business in malaria endemic areas and for the remaining 8 (8.9%) the birth place was unknown but they were not European citizens; for all the patients the visiting area is reported in Table 2. Sixty-nine patients (77.5%) were adults (age ≥ 18 years) and 20 (22.5%) were children (age < 18 years); 66 (74.2%) were males and 23 (25.8%) females (Table 2).

**Table 2:** Demographic data of the 89 malaria patients during January 2013–June 2017

Patients with malaria	Sex		Age		Area/Country visited		
	M	F	≥ 18 years	< 18 years	Africa^a^	Pakistan	India
73 *Pf*	52	21	58	15	73	–	–
1 *Pf* + *Po*	–	1	1	–	1	–	–
9 *Po*	9	–	8	1	9	–	–
2 *Pv*	2	–	–	2	–	1	1
1 *Pm*	1	–	1	–	1	–	–
1 *Pf* + *Pm*	1	–	1	–	1	–	–
2 *Pf* DNA	1	1	–	2	2	–	–

–: no cases

^a^Nigeria, Ivory Coast, Camerun, Guinea, Ghana, Burkina faso, Congo, Niger, Eritrea, Senegal, Somalia, Sudan, Togo, Mozambique, Ethiopia

The majority of these patients presented with fever, in particular 79 (88.7%) had fever at blood collection and 10 (11.2%) were without fever (Table 3). Seventy-one patients had no anti-malarial chemoprophylaxis (80.8%), 12 patients had incomplete chemoprophylaxis with mefloquine, 4 had complete chemoprophylaxis (3 with mefloquine and 1 with atovaquone + proguanil) and for the remaining 2 it was not known whether they had prophylaxis or not (Table 3).

**Table 3:** Clinical data of the 89 malaria patients during January 2013–June 2017

Patients with malaria	Fever		Prophylaxis		
	Fever pickup	No fever pickup	No	Yes (drug)	Unknown
73 *Pf*	67	6	59	10 (mefloquine incomplete) 3 (mefloquine)	1
1 *Pf* + *Po*	1	–	1	–	–
9 *Po*	8	1	5	2 (mefloquine incomplete) 1 (atovaquone + proguanil)	1
2 *Pv*	2	–	2	–	–
1 *Pm*	–	1	1	–	–
1 *Pf* + *Pm*	–	1	1	–	–
2 *Pf* DNA	1	1	2	–	–

–: no cases

For 85 out of the 89 patients with malaria the adopted therapy was known and it is detailed in Table 4 in relation to the plasmodial species detected and the parasitaemia range found. For 75 out of the 89 patients, a post-therapy follow-up was performed. For 37 patients a reduction in parasitaemia up to 0.001% was observed: in 16 cases after 48 h, in 11 cases after 72 h and in 10 cases after days. Full negativization was obtained in 1 case of *P. ovale* malaria after 72 h of quinine therapy. The distribution of symptoms and signs among patients with malaria and subjects without malaria is detailed in Table 5.

**Table 4:** Therapy of the 89 malaria patients during January 2013–June 2017

Parasitaemia range	Malaria species	Therapy	Follow-up (parasitaemia reduction up to 0.001%)			
			24 h	48 h	72 h	> 72 h
< 0.001–< 1%	40 *Pf*	12 Quinine^a^	–	1	1	1
		8 Atovaquone + proguanil	–	–	1	–
		14 Mefloquine	–	5	3	1
		6 Piperaquine tetraphosphate + dihydroartemisinin		1	–	–
	2 *Pv*	1 Chlorochine + primaquine	–	–	–	–
		1 Chlorochine + ceftriaxone	–	–	–	–
	9 *Po*	3 Mefloquine	–	2	–	–
		1 Quinine	–	–	–	–
		1 Chlorochine + primaquine	–	–	–	–
		1 Quinine + doxycycline + primaquine	–	–	1^d^	–
		1 Piperaquine tetraphosphate + dihydroartemisinin	–	–	–	–
		2 unknown	–	–	–	–
	1 *Pm*	1 Atovaquone + proguanil	–	–	–	1
	1 *Pf* + *Po*	1 Atovaquone + proguanil + primaquine	–	–	–	–
	1 *Pf* + *Pm*	1 Piperaquine tetraphosphate + dihydroartemisinin	–	–	–	–
1–10%	32 *Pf*	15 Quinine^b^	–	3	3	4
		9 Mefloquine	–	1	–	2
		4 Atovaquone + proguanil	–	1	2	–
		4 Piperaquine tetraphosphate + dihydroartemisinin	–	2	–	–
> 10% negative	1 *Pf*	1 Quinine	–	–	–	1
	2 *Pf*	2 Untreated^c^	–	–	–	–

–: no cases

^a^6 out of 12 were in association with doxycycline; ^b^ 5 out of 15 were in association with clindamycine, doxycycline, ampicillin + sulbactam or ceftriaxone; ^c^ in these cases, according to the Italian Ministry of Health guidelines, no therapy was administered because microscopy was negative; ^d^ a full negativization was obtained in this case

**Table 5:** Number of subjects (with and without malaria) with symptoms/signs related to malaria

Symptoms/signs	Subjects with malaria	Subjects without malaria
Gastrointestinal symptoms		
Diarrhoea	14	13
Abdominal pain	7	77
Nausea	6	6
Vomiting	17	12
Splenomegaly	5	3
Anaemia	20	3
Jaundice	19	1
Hepatomegaly	1	0
Kidney failure	1	0
Thrombocytopenia	63	2
Chills	26	18
Temperature > 37 °C	85	82
Sweating	5	3
Body pain	13	15
Cough	6	5
Pharyngodinia	3	6
Dispnea	2	6
Headache	28	18
Asthenia	10	6
Hypotension	1	0

For 4 (2%) and for 28 (14.1%) out of the overall 199 negative subjects 3 and 2 samples, respectively, were examined before excluding malaria diagnosis, while for the remaining 167/199 (83.9%) subjects only 1 sample was examined. In 38 out of these 167 subjects (22.7%) infections other than *Plasmodium* sp. have been found: 12 systemic infections (4 *Escherichia coli* + *Shigella* spp., 2 *Propionibacterium acnes*, 1 *Salmonella* sp., 1 *Escherichia coli*, 1 *Staphylococcus aureus*, 1 *Staphylococcus epidermidis*, 1 *Staphylococcus hominis*, 1 *Staphylococcus warneri*), 10 respiratory infections (2 *Adenovirus*, 2 *Haemophylus influenzae*, 1 *Streptococcus dysgalactiae*, 1 *Mycobacterium africanum*, 1 *Staphylococcus aureus* + *Enterobacter cloacae*, 1 *Escherichia coli*, 1 *Streptococcus pyogenes*, 1 *Mycobacterium tuberculosis*), 7 gastroenteritis (2 Norovirus, 2 *E. coli*, 1 *E. coli* + *Salmonella enteriditis*, 1 *Salmonella enteritidis* + *Candida albicans*, 1 *Escherichia coli* + *Pseudomonas aeruginosa*) 5 urinary infections and 4 chronic viral infections by Human Immunodeficiency Virus (HIV) or Hepatitis B Virus (HBV) or Hepatitis C Virus (HCV) viruses. In the remaining 129 subjects no infections were found.

Microscopic examination revealed the presence of *Loa loa* microfilariae in 1 blood sample negative for *Plasmodium* sp. and belonging to a patient for whom the clinical suspicion of malaria had been initially formulated.

## Discussion

Malaria is no longer endemic in Italy since 1970 when the WHO officially declared Italy malaria-free where nowadays is the most commonly imported disease [6, 20, 24, 25]. The risk of imported malaria depends on malaria endemicity at the destination, the number and the behavior (adherence to personnel protective measures) of the travellers to risk areas, adequate use of chemoprophylaxis, and efforts for vector control around the accommodation [26]. The incidence of imported malaria varies widely in non-endemic countries because of the variability in disease recognition, diagnostic capabilities, reporting protocols and adherence to those protocols. It is essential to have sensitive and specific malaria diagnostic tools to prevent over-treatment and the spread of disease [2] and it is also important to recognize that many hospital laboratories may not have microscopists with experience reading and interpreting blood films for malaria, particularly overnight or during the weekend [27].

In this study, the trend in malaria cases at the University Hospital of Parma, Italy, during January 2013–June 2017 is reported also with the treatment practices and the outcome of malaria cases; the study describe the comparison of the three diagnostic tests routinely used in our hospital to diagnose malaria cases: microscopy, ICT (BinaxNOW^®^Malaria) and Real-time PCR assays able to detect *P. falciparum*/*P. vivax*/*P. malariae*/*P. ovale curtisi/P. ovale wallikeri* and also *P. knowlesi*, even if in this study such DNA was not searched for because no patients were natives or came from Southeast Asia. Most of the analysed samples arrived at the laboratory from the Emergency Unit and the diagnosis was made by the methods above described following the flow chart adopted in this laboratory previously reported [6]. On holidays and nights only the search for absence or presence of *Plasmodium* sp. was performed by microscopic examination (after acridine orange and Giemsa staining) within 2 h from the arrival of the sample, in order to establish the presence or absence of *Plasmodium* sp. and to allow the administration of a prompt therapy in case of positivity (genus identification only). The following working day the result was confirmed by a parasitologist, including species identification, stages and parasitaemia.

The data reported in this study show that the malaria cases in the considered non-endemic setting have been increasing, from 20% observed in 2000 [6, 20] to a peak of 37.5% in 2015 likely as a result of the increase in the number of “forced migrants” (asylum seekers, refugees and torture victims) that are coming in recent years in addition to the tourists or business travellers to tropical regions, migrants visiting friends and relatives in their origin country as previously observed [6, 20, 28]. For some years, according to the Ministry of Health, all immigrants in Italy are submitted to mandatory health checks (tuberculosis, viral hepatitis, HIV). In particular, in the Emilia Romagna region, where this laboratory is located [29], a Public Health Office has established a special care service called “Immigrant Health Space”, to which such patients can address in case of clinical manifestations or pathologies. Generally, immigrants are less likely to take anti-malaria prophylaxis [30] compared to tourists or business travellers to endemic regions and this was observed also in this study where only 4 (4.5%), 3 Italians and 1 foreigner, of the 89 patients with malaria had correct anti-malarial chemoprophylaxis. As regards to the country of malaria acquisition, during the period considered in this study, the same picture of previous years [6, 20] was observed showing that the prevalence of malaria was especially due to immigrants, mostly from Africa except for one case from Pakistan and one from India confirming *P. falciparum* as the most frequently species detected and the occurrence of mixed infections (2) both involving *P. falciparum*.

The symptoms of malaria are non-specific: fever, observed in this study in the 95.5% of malaria patients when they presented to the Emergency Unit was in fact present also in the 92.1% of subjects without malaria as well as headache, present in the 31.4% of malaria patients and in the 20.2% of subjects without malaria. These symptoms with malaise are commonly the first manifestations. An important consideration is that up to half of patients may not febrile when they present to the Emergency Unit and that the classic symptoms of cyclic fevers, rigors, and cold sweats are often not present [27, 31] as also observed in this study where instead of the typical symptoms of chronic malaria including, in addition to fever (95.5%) and chills (29.2%), splenomegaly (5.6%), anaemia (20 cases out of the 23 for which such data were available) and jaundice (19 cases out of the 22 for which such data were available), and gastrointestinal problems such as diarrhoea (15.7% of 89 malaria cases), vomiting (19.1%), abdominal pain (7.8%), and nausea (6.7%) were the predominant. As expected, these symptoms occurs mostly in African patients who are repeatedly infected by *Plasmodium* sp., while in non-African patients they were not observed. It is important to note that even if in literature splenomegaly is observed in 24% to 40% of cases of uncomplicated malaria [31–33], in this study this was observed only in the 5.6% of malaria cases. As expected in malaria patients [27, 34], also in this study other non-specific symptoms/signs who may be related to malaria such as cough (6.7%), dyspnea (2.24%), headache (31.4%) and asthenia (11.2%) were detected.

Untreated, approximately 10% of uncomplicated imported malaria will progress to complicated or severe malaria defined as one or more of the following symptoms, occurring with *P. falciparum* asexual parasitaemia and without identified alternative causes: acidosis, hypoglycaemia, severe anaemia, jaundice, and hyperparasitemia [2]. Many factors influence the disease manifestations and the likelihood of disease progression to severe malaria and death [2], such as the *Plasmodium* species, the levels of innate and acquired immunity in the host, and the timing and efficiency of treatment, if any. WHO recommends artemisinin-based combination therapy (ACT) for the treatment of uncomplicated malaria caused by *P. falciparum* by combining 2 active drugs with different mechanisms of action [2]. ACT is the most effective antimalarial drugs available today and the choice should be based on the results of therapeutic efficacy studies against local strains of *P. falciparum* malaria. In order to prevent relapses of the hepatic hypnozoite form of *P. vivax* and *P. ovale*, primaquine is added and dose and frequency of the administration is guided by the patient’s glucose-6-phosphate dehydrogenase (G6PD) enzyme activity [2]. In this study, quinine has been the most widely used drug as first-line therapy (35.3%), followed by atovaquone + proguanil (16.1%), mefloquine (30.5%), piperaquine tetraphosphate + dihydroartemisinin (14.1%), chloroquine + primaquine (2.3%), and chloroquine with ceftriaxone (1.1%). Primaquine was used in all cases of non-*P. falciparum* malaria except that in a *P. ovale* positive patient due to lack of glucose-6-phosphate dehydrogenase (G6PD) enzyme activity. All drugs used in the study had the same efficacy and no chloroquine resistance was observed so that all the 89 malaria patients demonstrated a favourable outcome. No cases of severe malaria were observed although, in one patient, one of the WHO severe malaria criteria (parasitaemia > 10%, confirmed by 3 experienced microscopists) have been observed at the time of admission. All patients were discharged when a reduction of parasitaemia up to < 0.001% was observed, and this mostly occurred within the 3 days of therapy. The complete negativization after 72 h of therapy has been achieved only in 1 case and only in 14 out of the 73 *P. falciparum* cases gametocytes have been observed in the blood, a sign of a favorable evolution of the infection.

As concerns the comparison of the diagnostic assays used for the laboratory diagnosis of malaria, as expected, and already reported in the previous years [6, 20, 24], in this study molecular methods demonstrated to be more sensitive and specific than microscopy, both detecting additional cases of *P. falciparum* malaria and of mixed infections missed by microscopy and correctly identifying the 5 species of *Plasmodium* sp. of medical interest (*P. falciparum*, *P. vivax*, *P. ovale curtisi, P. ovale wallikeri*, *P. malariae*), respectively (*P. knowlesi* DNA was not searched for because no patients were natives or came from Southeast Asia). In fact, microscopy gave an incorrect diagnosis in the 1.12% of cases (mistaking *P. ovale* with *P. malariae*), likely due to the fact that these infections do not reach as high parasite levels as those by *P. falciparum*, as similarly observed in Parma from 2000 to 2012 [6]. Real-time PCR gave species identification in 3 out of 89 (3.37%) positive samples (1 *P. falciparum*, 2 *P. ovale*) in which plasmodial species had not been identified by microscopy, whose results were limited to genus identification (2 cases) and to *P. ovale*/*P. vivax* in the remaining case. In such cases, the incorrect identification of species by microscopy can be explained by the low parasitaemia (< 0.001–0.26%). As regards to RDTs, in this study BinaxNOW^®^, contrary to what has been described in the literature [10, 35], is found to be less sensitive than microscopy and Real-time PCR not revealing 6 infections; in 1 case by *P. ovale curtisi*, in 1 by *P. ovale wallikeri* and in 1 by *P. falciparum* + *P. malariae*. These results could be likely due to the low parasitaemia present in the samples, despite in 3 *P. ovale curtisi* cases the parasitaemia level was higher than that of other non-*P. falciparum* cases having a positive RDT result.

The plasmodial species most prevalently detected in the period included in the study was *P. falciparum*, followed by *P. ovale*, and then by *P. vivax* and *P. malariae*, showing the same picture of that observed in the period 2000–2012 [6]. Regarding *P. ovale*, it is notable that 3 out of the 9 *P. ovale* cases were caused by the *P. ovale wallikeri*, species detected in the blood of 3 patients coming from Mozambique, Ivory Coast and Niger, finding that confirms the presence and circulation of this *P. ovale* species also in Africa, and mostly in West Africa [6].

## Conclusions

This study shows an update and a thorough analysis of imported malaria cases in the area of Parma in the past 4 and a half years from the point of view of the total case management, clinical and diagnostic. The malaria cases in Parma are increasing (from 20% observed in 2000 [6, 20] to a peak of 37.5% in 2015) likely as a result of the increase in the number of “migrants”, almost all from Africa, that are coming in recent years in addition to the tourists or business travellers to tropical regions, migrants visiting friends and relatives in their origin country as previously observed. *Plasmodium falciparum* was the most frequently observed species and 2 mixed infections, both including *P. falciparum*, were detected. No fatal case was observed and the drug mostly used was the quinine. As expected, molecular methods demonstrated to be more sensitive and specific than microscopy, both detecting additional cases of *P. falciparum* malaria missed by microscopy and correctly identifying the 5 species of Plasmodium of medical interest, respectively. Regard to RDTs, in this study BinaxNOW^®^, contrary to what has been described in the literature was less sensitive than microscopy and Real-time PCR.

In conclusion, the data reported in this study may stimulate the clinicians in non-endemic areas to suspect malaria also in cases where the most typical symptoms (cyclic fevers, rigors, and cold sweats) are absent, and, on the other hand, the parasitologists to confirm the results of microscopy, still remaining the reference method, with molecular methods, even if they are more labour-intensive, in order to avoid misdiagnosis.
